# Mitochondrial-Derived Peptide MOTS-c Increases Adipose Thermogenic Activation to Promote Cold Adaptation

**DOI:** 10.3390/ijms20102456

**Published:** 2019-05-17

**Authors:** Huanyu Lu, Shan Tang, Chong Xue, Ying Liu, Jiye Wang, Wenbin Zhang, Wenjing Luo, Jingyuan Chen

**Affiliations:** Department of Occupational and Environmental Health and the Ministry of Education Key Lab of Hazard Assessment and Control in Special Operational Environment, School of Public Health, Fourth Military Medical University, No.169 West Chang-le Road, Xi’an 710032, Shaanxi, China; Lu_Huanyu@163.com (H.L.); Tangshan_fumm@163.com (S.T.); xuechong456@fmmu.edu.cn (C.X.); liuying2015@fmmu.edu.cn (Y.L.); wangjiye@fmmu.edu.cn (J.W.); zwb@fmmu.edu.cn (W.Z.)

**Keywords:** MOTS-c, adipose metabolism, thermogenesis, cold adaptation, browning fat

## Abstract

Cold exposure stress causes hypothermia, cognitive impairment, liver injury, and cardiovascular diseases, thereby increasing morbidity and mortality. Paradoxically, cold acclimation is believed to confer metabolic improvement to allow individuals to adapt to cold, harsh conditions and to protect them from cold stress-induced diseases. However, the therapeutic strategy to enhance cold acclimation remains less studied. Here, we demonstrate that the mitochondrial-derived peptide MOTS-c efficiently promotes cold adaptation. Following cold exposure, the improvement of adipose non-shivering thermogenesis facilitated cold adaptation. MOTS-c, a newly identified peptide, is secreted by mitochondria. In this study, we observed that the level of MOTS-c in serum decreased after cold stress. MOTS-c treatment enhanced cold tolerance and reduced lipid trafficking to the liver. In addition, MOTS-c dramatically upregulated brown adipose tissue (BAT) thermogenic gene expression and increased white fat “browning”. This effect might have been mediated by MOTS-c-activated phosphorylation of the ERK signaling pathway. The inhibition of ERK signaling disturbed the up-regulatory effect of MOTS-c on thermogenesis. In summary, our results indicate that MOTS-c treatment is a potential therapeutic strategy for defending against cold stress by increasing the adipose thermogenesis via the ERK pathway.

## 1. Introduction

Cold temperatures are a common feature of hostile environments, such as high-altitude and polar regions. Local residents, occupational workers, and military personnel exposed to these conditions suffer from cold-induced injury and disease. As reported, exposure to cold temperature increases the risk of freezing injuries and systemic or accidental hypothermia, impairs cognitive performance, and triggers cardiovascular deaths [1,2,3]. Cold acclimation is a potential strategy to allow adaptation to cold environments and it attenuates cold-induced impairments [4]. Prolonged or intermittent repeated exposure to low temperatures is a way to improve cold acclimation and enhance the ability of cold adaptation [2,5,6]. However, the therapeutic strategy to increase the ability of cold adaptation is still less studied.

Non-shivering thermogenesis is a critical characteristic of cold acclimation and plays a key role in facilitating cold adaptation [7]. Brown and browning white fat are a major source of heat production from non-shivering thermogenesis [8,9]. Indeed, fat tissue can be divided into brown, white, and brown-like adipose. Brown adipose tissue (BAT) is highly vascularized and enriched with mitochondria and multilocular lipid droplets. This feature enables the BAT to rapidly burn intracellular lipid droplets and TG-derived FA from plasma and to produce heat via uncoupling protein-1 (UCP1)-mediated thermogenic energy dissipation [10]. White adipose tissue (WAT) is different from BAT at functional, morphological, and molecular levels. WAT contains a characteristic unilocular lipid droplet and stores excess energy as triacylglycerols [11]. Further, brown-like adipocytes are identified in the WAT of rodents and humans [12,13]. These cells, with a multilocular morphology and expressing the brown adipocyte-specific UCP1, have a similar thermogenic function as BAT. As reported, cold exposure efficiently triggers brown and brown-like fat non-shivering thermogenesis [14]. Therefore, increasing adipose thermogenic capacity and activity is a potential therapeutic strategy to facilitate cold adaptation.

Mitochondria are the main organelle for cellular thermogenic metabolism and energy production. Of interest, mitochondria process an independent genome (mtDNA) and a unique genetic code to encode 37 genes. MOTS-c is identified as a mitochondrial derived peptide originating from the mtDNA and can be secreted into the blood, suggesting it functions in cell-autonomous and hormonal ways [15,16,17,18]. According to recent reports, MOTS-c has been shown to target the skeletal muscle and enhance glucose metabolism [19]. Our previous works have demonstrated that MOTS-c inhibited osteoclastogenesis to prevent ovariectomy-induced bone loss [20]. MOTS-c also regulated adipose metabolic homeostasis and increased brown fat activation to defend against ovariectomy-induced obesity [21]. However, it is still unclear whether MOTS-c can control adipose thermogenesis under normal conditions, and the effects of MOTS-c on acute cold-induced thermogenesis versus cold acclimation have not been studied, nor have the effects on BAT thermogenic capacity and WAT browning been evaluated in detail.

In this study, we observed that the level of MOTS-c reduced in serum following chronic cold exposure. MOTS-c administration efficiently maintained relative higher body temperature and promoted the ability of cold adaptation, especially upon acute cold stimuli. MOTS-c administration increased brown fat activation and white fat “browning.” Thus, our data present a more thorough and convincing analysis of the MOTS-c effect on adipose thermogenic function and indicate that MOTS-c administration is a potential therapeutic strategy to enhance cold adaptation and prevent cold-induced disease.

## 2. Results

### 2.1. MOTS-c Administration Promotes the Ability of Cold Adaptation

**Figure 1:**
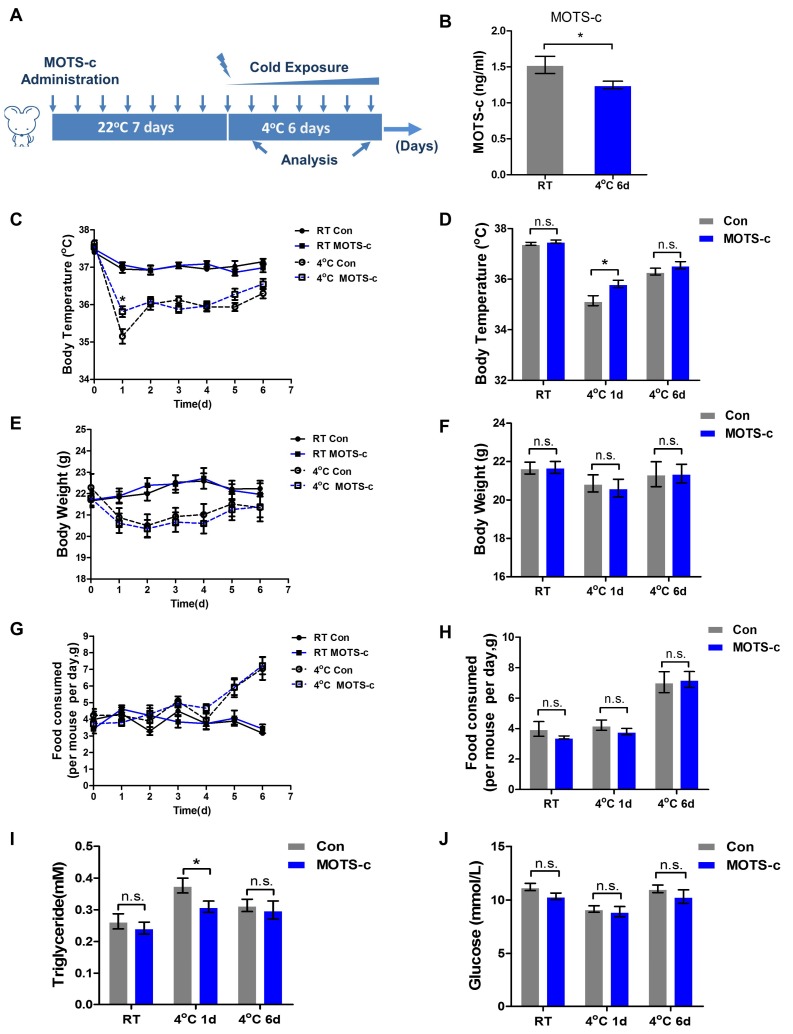
MOTS-c administration increases the ability of cold adaptation upon acute cold exposure. (**A**) Experimental design for the study in MOTS-c administration upon cold exposure. (**B**) The serum level of MOTS-c following 4 °C exposure and the standard curve and 450 nm absorption signaling are shown (*n* = 8 per group). (**C**,**D**) Body temperature during cold exposure and the change in body temperature was analyzed at the first and sixth days of cold exposure. (*n* = 8 per group). (**E**,**F**) Body weight during cold exposure and the change in body weight was analyzed at the first and sixth days of cold exposure (*n* = 8 per group). (**G**,**H**) Food intake during cold exposure and the change in food intake was analyzed at the first and sixth days of cold exposure. (*n* = 8 per group). (**I**,**J**) The serum levels of triglycerides and glucose were detected at the first and sixth days of cold exposure. (*n* = 8 per group). All data are represented as mean ± SEM. Differences between the two groups were determined by a two-tailed Student’s *t*-test. * *p* < 0.05, n.s. = not significant.

Firstly, we detected the content of MOTS-c using MOTS-c-specific ELISA. Notably, chronic cold exposure (six days) lowered the level of MOTS-c in serum (Figure 1B). However, upon acute cold exposure (one day), the circulating MOTS-c level changed without statistical significance (Figure S1A). Considering that MOTS-c is a bioactive peptide that affects body metabolic status, we administrated 5 mg/kg MOTS-c by intraperitoneal injection, and the dosage of MOTS-c used in this study was strictly referred to our previous published work [19,21]. After a week of administration, mice were transferred to a 4 °C environment and continually administered 5 mg/kg MOTS-c. During cold exposure, body temperature, body weight, and food intake were daily measured. The results showed that acute cold exposure reduced body temperature and body weight. The MOTS-c-administered mice maintained a relatively higher body temperature compared to the control mice, especially on the first day of acute cold exposure (Figure 1C,D). Following six days of chronic cold exposure, body temperature, body weight, and food intake were gradually increased, but there were no significantly different changes between the two groups (Figure 1C–H). Next, we tested the serum lipid and glucose levels in the two groups. Upon the first day of acute cold exposure, serum triacylglycerol (TG) concentration increased and MOTS-c administration significantly lowered the level of TGs (Figure 1I). However, after six days of chronic cold exposure, the serum TG concentration between the two groups had not significantly changed (Figure 1I). Additionally, we did not find significant changes in the level of glucose between the two groups during cold exposure (Figure 1J). These findings suggest that MOTS-s administration affected lipid metabolism and enhanced the ability of cold adaptation upon acute cold exposure.

### 2.2. MOTS-c Administration Prevents Acute Cold-Induced Liver Lipid Deposition

**Figure 2:**
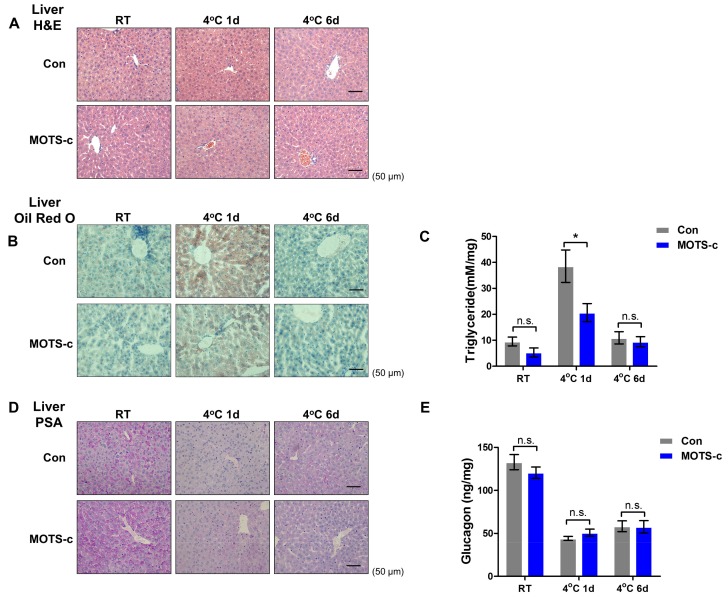
MOTS-c administration alleviates acute-cold-induced hepatic lipid accumulation. (**A**) H&E staining of the liver showing the morphologic change (scale bar = 50 μm). (**B**) Oil Red O staining of the liver showing the lipid droplets (scale bar = 50 μm). (**C**) Triglycerides content in the liver (*n* = 8 per group). (**D**) PAS staining of the liver presenting the relative level of glycogen (scale bar = 50 μm). (**E**) The glycogen content in the liver (*n* = 8 per group). All data are represented as mean ± SEM. Differences between the two groups were determined by a two-tailed Student’s *t*-test. * *p* < 0.05, n.s. = not significant.

Considering the higher level of serum lipid after acute cold exposure, we tested and analyzed the morphology and metabolic functional changes in the liver. Firstly, H&E staining showed that there was no dramatically morphologic change in livers between the two groups during cold exposure (Figure 2A). Furthermore, Oil Red O staining showed that lipid droplets were drastically enriched in the liver, and MOTS-c administration markedly lowered lipid accumulation upon acute cold exposure (Figure 2B). However, following six days of chronic cold exposure, lipid droplets in the liver decreased to a normal level, and there was no difference between the two groups. The TG content analysis showed a similar result (Figure 2C). In addition, PSA staining showed that hepatic glycogen content declined during cold exposure, and the glycogen ELISA analysis indicated a similar result (Figure 2D,E). However, MOTS-c administration did not change this phenomenon. From the Lee et al. study [19], microarray analyses from HEK293 cells treated with MOTS-c displayed that MOTS-c had a significant effect on functional pathways related to metabolic signaling and remarkably influenced lipid metabolism (Figure S1B–D). These findings suggest that MOTS-c is involved in controlling liver lipid metabolism without affecting glycogen metabolism.

### 2.3. MOTS-c Administration Increases the White Fat “Browning”and Brown Fat Activation upon Acute Cold Exposure

**Figure 3:**
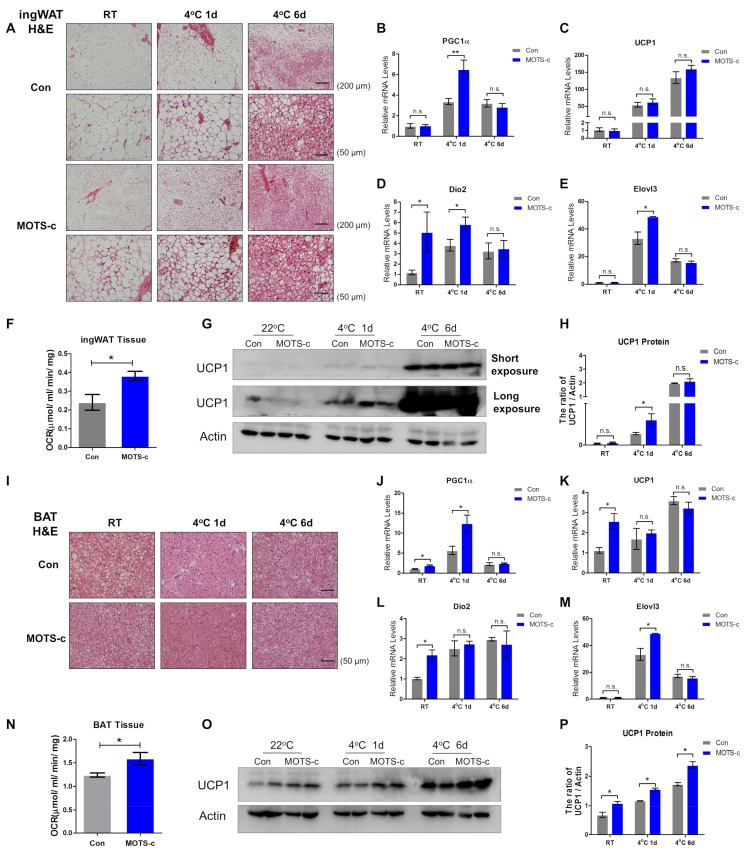
MOTS-c administration promotes white fat browning and Brown Fat Activation during cold exposure. (**A**) H&E staining of ingWAT to show the morphologic change during cold exposure (scale bar = 200 and 50 μm). (**B–E**) The mRNA levels of genes for thermogenesis (PGC1α, UCP1, Dio2, and Elovl3) on the first and sixth days of cold exposure (*n* = 4, per group). (**F**) Total oxygen consumption per gram in ingWAT from normal saline (Con) and MOTS-c treated mice under basal conditions were measured using a Clark electrode. (*n* = 3, per group). (**G**,**H**) The protein level of UCP1 detected by Western blot and immunoreactive bands were quantified by densitometric analysis using ImageJ (*n* = 4, per group). (**I**) H&E staining of BAT to present the morphologic change during cold exposure (scale bar = 50 μm). (**J–M**) The mRNA levels of genes for thermogenesis (PGC1α, UCP1, Dio2, and Elovl3) in BAT (*n* = 4, per group). (**N**) Total oxygen consumption per gram in BAT from normal saline (Con) and MOTS-c treated mice under basal conditions were measured using a Clark electrode. (*n* = 3, per group). (**O**,**P**) The protein level of UCP1 detected by Western blot and immunoreactive bands were quantified by densitometric analysis using ImageJ (*n* = 4, per group). All data are represented as mean ± SEM. Differences between the two groups were determined by a two-tailed Student’s *t*-test. * *p* < 0.05, ** *p* < 0.01, n.s. = not significant.

Next, considering adipose tissue is critical to affecting the lipid metabolic status, we examined whether MOTS-c functioned in adipose metabolism. White adipose H&E staining showed that a denser structure gradually emerged, and the unilocular intracellular lipid droplet turned into multilocular lipid droplets during cold exposure, suggesting more brown-like adipocytes exist in white fat (Figure 3A). Of interest, MOTS-c administration reduced the size of lipid droplets under normal conditions. Upon acute cold exposure, MOTS-c administration promoted more multilocular lipid droplets, indicating that MOTS-c increased white fat “browning” (Figure 3A). To further clarify this phenomenon, RT-PCR analysis showed that, except for Dio2, the RNA levels of thermogenic genes (PGC1α, UCP1, and Elovl3) did not significantly change in MOTS-c-administered mice under normal conditions. Moreover, MOTS-c administration upregulated the RNA levels of genes for thermogenesis upon the first day of cold exposure, but had no effect on the RNA levels of thermogenic genes following six days of cold exposure (Figure 3B–E). Primers used in the study were shown in Table 1. Furthermore, ingWAT from mice treated with MOTS-c presented a higher total oxygen consumption rate under basal conditions measured by Clark electrode (Figure S2A and Figure 3F). Consistent with this, Western blot results showed that six days of chronic cold exposure dramatically triggered a higher level of UCP1, and MOTS-c administration rapidly increased the level of UCP1 on the first day of cold exposure without significantly changing the level of UCP1 after six days of cold exposure (Figure 3G,H). Thus, MOTS-c administration promoted white fat “browning” upon acute cold exposure.

Brown adipose H&E staining showed that brown fat administered with MOTS-c displayed a denser structure and a lower level of lipid droplets under normal conditions, indicating that MOTS-c administration promoted brown fat activation (Figure 3I). Next, RT-PCR analysis observed that MOTS-c upregulated the levels of thermogenic genes (PGC1α, UCP1, and Dio2) under normal conditions. Upon the first day of cold exposure, MOTS-c also greatly increased the RNA levels of PGC1α and UCP1. However, following six days of chronic cold exposure, there was no significant difference in RNA levels between the two groups (Figure 3J–M). And under room temperature, BAT from mice treated with MOTS-c presented a higher total oxygen consumption rate (Figure S2A and Figure 3N). Furthermore, different from the RT-PCR data, Western blot results showed that MOTS-c administration continually significantly enhanced the expression of UCP1 during cold exposure (Figure 3O,P). We concluded that MOTS-c administration promoted brown fat activation.

### 2.4. MOTS-c Improves the Expression of Thermogenic Genes In Vitro

**Figure 4:**
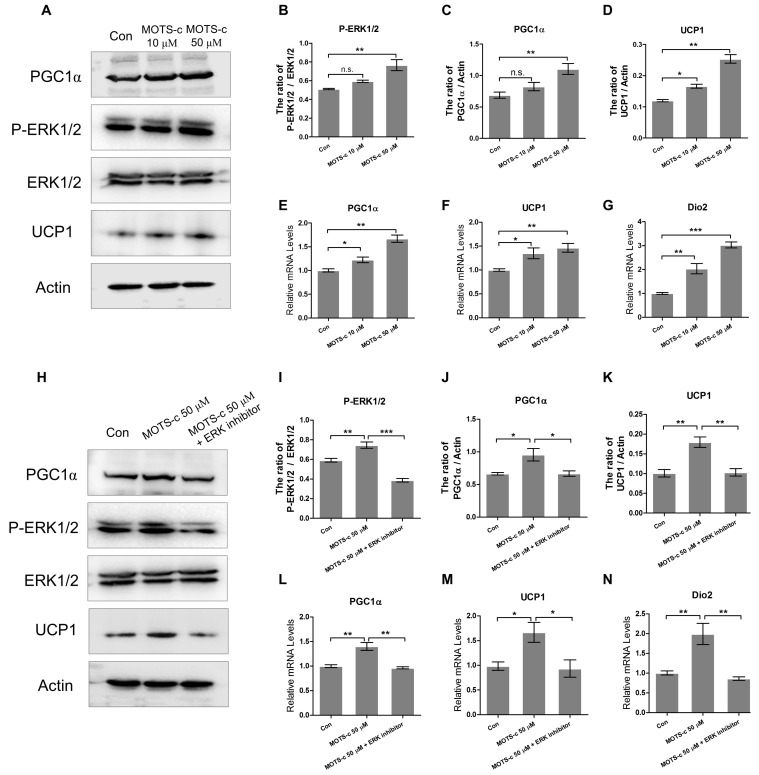
MOTS-c triggers thermogenic genes program via the ERK signaling pathway. (**A**–**D**) The protein levels of phospho-ERK1/2, PGC1α, and UCP1 in adipocytes treated with MOTS-c and immunoreactive bands were quantified by densitometric analysis using ImageJ (*n* = 4, per group). (**E**–**G**) The RNA level of thermogenic genes (PGC1α, UCP1, and Dio2) (*n* = 4, per group). (**H**–**K**) The level of phospho-ERK1/2, PGC1α, and UCP1 in adipocytes treated with MOTS-c and ERK inhibitor, and immunoreactive bands were quantified by densitometric analysis using ImageJ (*n* = 4, per group). (**L**–**N**) The RNA level of thermogenic genes (PGC1α, UCP1, and Dio2) (*n* = 4, per group). All data are represented as mean ± SEM. Differences between the two groups were determined by a two-tailed Student’s t-test. * *p* < 0.05, ** *p* < 0.01, *** *p* < 0.001, n.s. = not significant.

In an in vitro study, we used adipose stromal vascular fraction cells (SVFs) to differentiate mature adipocytes. After differentiation, cells were treated with different dosages of MOTS-c peptide. Firstly, Western blot result showed that the protein levels of phosphorylated ERK (P-ERK), PGC1α, and UCP1 were elevated after MOTS-c treatment (Figure 4A–D). Additionally, RT-PCR data showed that MOTS-c treatment significantly raised the RNA levels of PGC1α, UCP1, and Dio2 in vitro (Figure 4E–G). To further examine the possible involvement of the ERK kinase pathway in MOTS-c-induced thermogenic effects, adipocytes were treated with or without an ERK inhibitor (ERK1/2 inhibitor 1) in the presence of MOTS-c. ERK phosphorylation induced by MOTS-c was seriously suppressed by the ERK inhibitor, whereas there was no reduction in the amount of total ERK protein. And the high protein levels of PGC1α, and UCP1 triggered by MOTS-c were blocked by ERK inhibitor (Figure 4H–K). Furthermore, the ERK inhibitor treatment abolished the increasing MOTS-c-mediated expression of PGC1α, UCP1, and Dio2 mRNA, indicating that the MOTS-c-induced thermogenic genes program was mediated via activation of the ERK signaling pathway (Figure 4L–N).

## 3. Discussion

In this study, we observed a process from metabolic stress to metabolic adaptation during cold exposure. As we showed, acute cold exposure significantly triggered a lower body temperature and a reduction in body weight accompanied by elevated triglyceride and hepatic lipid accumulation. However, following chronic cold exposure, body temperature and body weight maintained relatively high levels, and serum triglyceride and hepatic lipid content mostly returned to normal. According to these findings, we considered that a rapid promotion of cold adaptation can alleviate acute cold-induced metabolic stress.

As recently reported, thermogenic fat-consuming energy during cold exposure is critical to keeping body temperature homeostasis and sustaining lipid metabolic homeostasis [8]. Firstly, brown adipose tissue (BAT) is vital for non-shivering thermogenesis during cold exposure in rodents and humans. Brown adipose tissue (BAT) expends circulating glucose and fatty acids for UCP1-mediated heat production to defend against hypothermia [10,22]. Recent studies have shown an inverse relationship between BAT activity and shivering. Enhanced BAT activity contributes to an increase in total energy expenditure upon acute cold exposure [8,23]. Next, brown fat activity favors beneficial lipid metabolism during cold exposure. As reported, acute cold exposure immediately induces transient hyperlipidemia and triggers brown fat metabolic activation, which efficiently uptakes fatty acids to control plasma triglyceride clearance and blood lipid abundance, due to its thermogenic program [10,24]. Of interest, in addition to increasing heat production, BAT activation improves the deleterious effects of transient hyperlipidemia and prevents lipid ectopic accumulation. Furthermore, cold exposure is associated with high cardiovascular risk. Cold-induced lipolysis significantly increased the plasma levels of small low-density lipoprotein (LDL) remnants, leading to atherosclerotic plaque growth by increasing lipid deposition. BAT activation by cold exposure improves cholesterol metabolism, accelerates the hepatic clearance of the cholesterol-enriched remnants, and protects from atherosclerosis [25,26]. Therefore, the therapeutic target to BAT is efficient in increasing cold adaptation and reducing the risk of cold-mediated diseases.

Recent data also show that there are two distinct types of brown fat: Classical brown fat and browning white fat, also called beige fat. Beige fat is a distinct type of UCP1-positive thermogenic cells emerged in white fat. Indeed, beige fat resembles white fat in having a low basal level of UCP1. However, similar to classical brown fat, cold exposure-induced high UCP1 expression and thermogenic rates in beige fat, which consumes circulating glucose and fatty acids in non-shivering thermogenesis [27]. Thus, regulating this ‘‘browning’’ effect by a therapeutic approach is essential for controlling metabolic homeostasis and enhances the ability of cold adaptation.

Of particular interest are peptides capable of fat thermogenesis. MOTS-c is a 16-amino acid peptide encoded from 12S rRNA region of the mitochondrial DNA and can be secreted into plasma as a thermogenic hormone. Previous studies have proved that MOTS-c targets to skeletal muscle and regulates insulin sensitivity in mice. MOTS-c treatment efficiently improves high-fat-induced hyperinsulinemia and hepatic lipid accumulation [17,19]. Notably, MOTS-c treatment also increases energy expenditure with significantly more heat production, but its mechanism is still unclear. In our previous work, we have clarified that MOTS-c effectively regulates adipose metabolism by decreasing fat deposition, increasing brown fat activation, attenuating inflammation invasion, and preventing ovariectomy-induced obesity [21]. Combined with these findings, we have suggested that MOTS-c is an adipose thermogenic activator for metabolic benefits. In our experiment before, we had treated mice with normal saline (Con) and MOTS-c (5 mg/kg, per day) for long term (45 days) under room temperature, and PET/CT imaging result showed that the PET signals were detected higher in the BAT and WAT position of the MOTS-c-treated mice (Figure S2A–C). Furthermore, we isolated BAT and ingWAT from mice treated with normal saline (Con) and MOTS-c. Then, total oxygen consumption per gram in BAT and ingWAT under basal conditions were measured using a Clark electrode. And the data presented that MOTS-c treatment increased the BAT and ingWAT total oxygen consumption rates (Figure 3F,N). Altogether, our finding indicated that MOTS-c has a greater capacity to increase the activity of BAT and “browning” WAT in mice under room temperature. However, it is still unclear whether MOTS-c has an effect on adipose thermogenic function following acute and chronic cold exposure. MOTS-c has been detected in various tissues, as well as in circulation in human and rodent plasma, and fasting has been shown to reduce endogenous expression of MOTS-c in certain metabolically active and mitochondrial rich tissues, as well as in plasma. In this study, our data showed that cold exposure lowered the level of circulating MOTS-c. Unfortunately, without a commercial MOTS-c antibody, changes in the endogenous level of MOTS-c in metabolic tissues from mice cannot be accurately detected. Considering that MOTS-c is an endocrine signal regulating facultative thermogenesis, we suggest that a lower level of circulating MOTS-c may be derived from the absorption and depletion of metabolically active tissues following chronic cold exposure (six days). But, upon acute cold exposure (one day), the circulating MOTS-c level altered without statistical significance. As reported, chronic cold exposure gradually alters the body metabolic status and well increases cold adaption, and acute cold exposure induces body metabolic stress and influences peripheral metabolic tissues [2,5,7,28]. Therefore, we suggest that circulating MOTS-c may be absorbed or degraded by the peripheral metabolic active tissues following chronic cold exposure, and this may explain that circulating MOTS-c level unchanged upon acute cold exposure. Finally, our study showed that MOTS-c administration enhances cold adaptation and presents a more thorough and convincing analysis of BAT and WAT thermogenesis under acute and chronic cold exposure conditions.

MOTS-c has been shown to play metabolic roles dependent on AMPK activation, and recent work shows that MOTS-c can translocate to the nucleus to improve metabolic stress by regulating nuclear gene expression [29]. Microarray analyses from HEK293 cells treated with MOTS-c for 72 h showed that MOTS-c is a metabolic active peptide (Figure S1B). MOTS-c had a significant effect on functional pathways related to metabolic and inflammatory signaling (Figure S1C). In the metabolic pathway, MOTS-c remarkably influenced lipid metabolism (Figure S1D). Consistent with this finding, our work showed that MOTS-c attenuated cold-induced serum elevated TGs and hepatic lipid accumulation. Additionally, we observed that, during chronic cold exposure, MOTS-c unaltered the UCP1 mRNA level, but upregulated its protein level, indicating that MOTS-c is involved in translational regulatory of UCP1 expression. Furthermore, our data showed that MOTS-c stimulated thermogenic gene expression via activation of the ERK pathway [30]. However, our previous work showed that this peptide enhanced the bactericidal capacity of macrophages and MOTS-c inhibited the phosphorylation mitogen-activated protein kinases (MAPKs) in macrophages [31]. Considering the ERK signal pathway involved in adipose thermogenesis, we suggest that the effects of MOTS-c treatment on thermogenic gene expression may depend on cell membrane receptors to mediate this mechanism. Due to the different types and distribution of cell membrane receptors in different cell types, the effect of MOTS-c on the MAPK signaling pathway may be inconsistent in adipocytes and macrophages. Since G-protein-coupled receptors (GPCRs) play a vital role in signal transduction in adipocytes (for example, β-adrenergic and adenosine receptors activate cAMP signaling and UCP1-dependent thermogenesis) [32,33], we considered whether the effect of MOTS-c was dependent on GPCRs. Similar to MOTS-c, humanin is a 24 amino acid peptide that is encoded in the 16S rRNA gene within the mitochondrial genome. Meanwhile, humanin is identified as a neuroprotective factor using the G protein-coupled formylpeptide receptor-like-1, and humanin treatment increases the phosphorylation of MAPK p44/42 (ERK 1/2), AKT, and STAT3 pathway [34]. Therefore, we speculate that MOTS-c can act through the G protein-coupled receptors to activate AKT, ERK1/2, and STAT3 signaling pathways. Approximately 25% of currently marketed drugs target GPCRs [35], thus illustrating the functions of MOTS-c on GPCRs is very important in disease and therapeutics. Our further work will explore this in greater detail.

**Figure 5:**
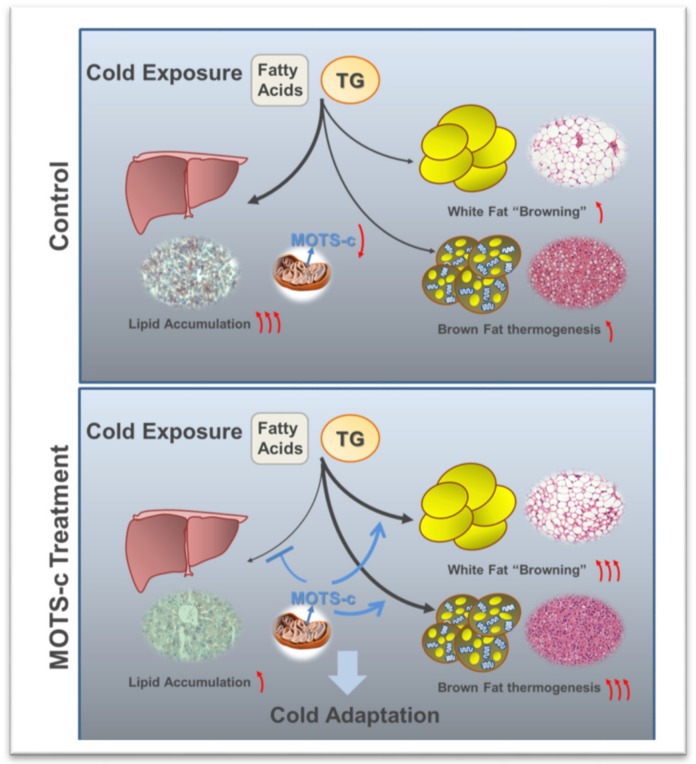
The effect of MOTS-c on lipid metabolism upon cold exposure. MOTS-c administration increases BAT activation and WAT browning to produce heat. And MOTS-c promotes lipid consumption and alleviates acute cold exposure induced elevated TGs and lipid accumulation in serum and liver. Black arrow: TGs and lipid absorbed by liver or BAT and WAT; Blue arrow: the effect of MOTS-c on lipid metabolism and metabolic organs; Red arrow: The change of MOTS-c, lipid content and thermogenic functions.

MOTS-c-mediated non-shivering thermogenesis may be a key contributor to increased energy expenditure. MOTS-c administration increased cold adaptation to defend against hypothermia, and alleviated acute cold exposure induced elevated TGs and lipid accumulation (Figure 5).

In summary, our study clarified that the mitochondrial derived peptide, MOTS-c, is an activator of adipose thermogenesis and suggested that it is a potential preventive drug to reduce the risk of cold-induced diseases.

## 4. Materials and Methods

### 4.1. Experimental Groups

C57BL/6 male mice (8~10 weeks old) were purchased from the Experimental Animal Center of Air Force Medical University. The mice were housed at the animal care facility at 22 °C with 12 h light/dark cycles. All mice were randomly divided into four groups (*n* = 8 mice per group): Control (injection with normal saline), MOTS-c administration (injection with 5 mg/kg MOTS-c per day), cold exposure (injection with normal saline), and cold exposure administrated with MOTS-c (injection with 5 mg/kg MOTS-c per day). The dosage used in this study refers to the published articles [15,16]. The mice were intraperitoneally (i.p.) injected with MOTS-c or normal saline every day for a week. Next, mice were individually housed in cages kept at 4 °C for cold exposure and continually treated daily with MOTS-c (Figure 1A). During cold exposure, body temperature, body weight, and food intake were monitored every day. Body temperature was measured using a rectal probe (Yellow Spring Instruments, Schaumburg, IL, USA) [36]. Following cold exposure, mice were euthanized with excess amounts of pentobarbitone. The animal experiments were approved by the Institutional Animal Care and Ethics Committee of Air Force Medical University (No.20180821, 9 September 2018).

### 4.2. Synthesis of Peptides

MOTS-c was synthesized by Sangon Biotech Company in China and was identified as having >98% purity by high-performance liquid chromatography. The amino acid sequence of MOTS-c was as follows: Met Arg Trp Gln Glu Met Gly Tyr Ile Phe Tyr Pro Arg Lys Leu Arg. The peptides were dissolved in ddH_2_O and frozen at −80 °C for no longer than a month until they were used.

### 4.3. Serum Parameters Analysis

Blood samples were collected in 1.5 mL blood collection tubes. The collected blood was allowed to stand at room temperature for 30 min, then 3000× *g* centrifuged for 10 min, and the supernatant was taken and stored at −80 °C. The level of triglyceride was detected using commercially available kits (Biovison K622, Milpitas, CA, USA) according to the manufacturer’s instructions. The level of glucose was tested using an Accu-Chek Active blood glucose meter. The level of MOTS-c was measured by a MOTS-c peptide enzyme immunoassay kit purchased from Peninsula Laboratories International, Inc. (San Carlos, CA, USA) (No. S-1518). According to plot data, the equation to calculate results was shown as—f(*x*) = a*exp(b*x) + c*exp(d*x), Coefficients (with 95% confidence bounds): a = 2.524 (2.091, 2.956); b = −1.1 (−1.668, −0.532); c = 0.6561 (0.3107, 1.002); d= −0.003566 (−0.01442, 0.007293). Goodness of fit: SSE:0.0447, R-square: 0.9945; Adjusted R-square: 0.989, RMSE: 0.1222.

### 4.4. H&E (Hematoxylin–Eosin) Staining

Adipose and liver tissues were immediately fixed in 4% paraformaldehyde and embedded in paraffin. Ten-micrometer sections were stained with H&E. Briefly, sections were stained in hematoxylin dye for 3~5 min. After washing, sections were dehydrated in alcohol successively for 5 min, and stained in eosin dye for 5 min. Sections were then sealed and observed under the microscope.

### 4.5. Liver Glycogen Staining and Glycogen Content Analysis

For PAS staining (Periodic Acid-Schiff stain, Wuhan, China), liver tissues were fixed with 4% paraformaldehyde, embedded in paraffin, and sliced. Next, the sections were stained in periodate dye solution for 15~20 min. After washing, sections were stained with Schiff dye in the dark for 30 min. Sections were then stained in hematoxylin dye for 3–5 min. For glucagon content analysis, liver tissue (~100 mg) was homogenized in 500 μL of RIPA lysis buffer (Beyotime P0013B, Shanghai, China) and tested by Glucagon ELISA kit (4A Biotech Co., Ltd., CSPE0012, Beijing, China) according to the manufacturer’s instructions

### 4.6. Liver Oil Red O Staining and Triglyceride Content Analysis

For the liver tissue, fresh tissues were frozen and sliced, and sections were stained with a freshly prepared Oil Red O working solution for 20 min. After staining, the phenotype was observed by microscopy in visible light. For triglyceride (TG) content analysis, liver tissue (~100 mg) was homogenized in a 1 mL solution containing 5% NP-40 in water and diluted 10-fold with dH_2_O for the assay using commercially available kits (Biovison K622, Milpitas, CA, USA) according to the manufacturer’s instructions.

### 4.7. Cell Culture and Adipocyte Differentiation

For preadipocytes (stromal vascular fractions, SVF) isolation, inguinal subcutaneous fat from 5 mice was minced with scissors, digested with DMEM containing 0.1% collagenase type 1 and 2% bovine serum albumin. Digests were centrifuged, resuspended in DMEM with 10% fetal bovine serum, and transferred through a 40 mm nylon strainer onto plates. For adipocyte differentiation, SVF preadipocytes were allowed to reach confluence and cultured with a stimulation differentiation medium consisting of growth media supplemented with 0.5 mM 3-isobutyl-1-methyl-xanthine, 10 μM dexamethasone, 10 µg/mL insulin, and 5 μM rosiglitazone. After 2 days in a stimulation medium, cells were placed in a post-stimulation medium containing DMEM, 10% FBS, 5 µg/mL insulin, 2 nM T3 and 1 μM of rosiglitazone. The medium was changed every 2 days. Cells were cultured in a differentiation medium on Day 6 and treated with MOTS-c (10 μM, 50 μM). After 48 h of treatment, cells were collected for analysis. To test the MOTS-c ERK-dependent role, adipocytes were co-cultured with MOTS-c and ERK inhibitor (10 nM, ERK1/2 inhibitor 1, MedChemExpress, HY-112287, Monmouth Junction, NJ, USA).

### 4.8. RNA Isolation and Quantitative RT-PCR

Total RNA was isolated using TRIzol (Invitrogen, Carlsbad, CA, USA) and reverse-transcribed using a High-Capacity cDNA Reverse Transcription kit (Takara, Kusatsu, Japan). The complementary DNA (cDNA) was analyzed by quantitative PCR with reverse transcription (qRT–PCR). Briefly, 20 ng cDNA and 100 nmol of each primer were mixed with SYBR GreenER qPCR SuperMix (Takara). Reactions were performed in a 96-well format using the 7900HT Fast Real Time PCR System (Applied Biosystems, Foster City, CA, USA). The relative abundance of mRNA was normalized to 36B4 mRNA as the invariant control. The gene expression profile (GSE65068) was analyzed, and significant genes were selected by a fold change >1.5, as well as a *p*-value <0.05. The data are shown in the Supplementary Materials.

### 4.9. Western Blot Analysis

Protein was extracted from BAT, ingWAT, and cells using a RIPA buffer (Beyotime Products, Beijing, China) containing a complete protease inhibitor cocktail (Roche, San Diego, CA, USA). For Western blot analysis, an equal amount of protein sample was loaded in each well of a 12% SDS-PAGE. After electrophoresis, proteins were transferred to PVDF membranes, blocked for 2 h with 5% BSA at RT, and blotted using UCP1 (Abcam ab10983, 1:1000, Abcam, Cambridge, UK), Actin (Sigma A1978, 1:1000), anti-ERK1/2 (Cell Signaling Technology #4695, 1:1000, Boston, MA, USA), and anti-Phospho-ERK1/2 (Cell Signaling Technology #4370, 1:1000, Boston, MA, USA) overnight at 4 °C. After that, the membranes were washed with TBST and incubated with HRP-conjugated secondary antibodies for 1.5 h at RT. Immune complexes were then detected using the ECL method and immunoreactive bands were quantified by densitometric analysis using ImageJ.

### 4.10. Micro PET/CT Imaging

PET/CT imaging was performed on Department of Nuclear Medicine, Xijing Hospital, Fourth Military Medical University. Mice were treated with normal saline (Con) and MOTS-c (5 mg/kg, per day) for long term (45 days) under room temperature, then mice were allowed to fast six hours and were anesthetized with isoflurane followed by a tail vein injection of 18F-FDG (120 mCi). Thirty mins after the injection of the 18F-FDG, the mice were subjected to PET/CT image test and analysis according to Wang Z. et al. [37].

### 4.11. Tissue Oxygen Consumption Test

Tissue oxygen consumption analysis was performed using a Clark electrode (Strathkelvin Instruments, North Lanarkshire, Scotland). Freshly BAT and ingWAT was isolated from normal saline (Con) and MOTS-c mice (*n* = 3, per group), rinsed in sterile saline, weighed, minced, and placed into respiration buffer (DMEM + 1% BSA). Readings were taken from three separate pieces of tissue of equivalent size. Oxygen consumption was normalized to tissue weight.

### 4.12. Statistical Analysis

All data are expressed as the mean ± standard error of the mean (SEM). The differences between the two groups were analyzed using a two-tailed Student’s *t*-test, and for multiple-group experiments, one-way ANOVA was used. The analysis was performed using Microsoft Excel and/or Graph Pad Prism. *p*-values < 0.05 were considered statistically significant, as indicated by asterisks in the figure legends. The sample size was estimated by pilot experiments that showed the trends of effects and their size.

## Figures and Tables

**Table 1:** The following primers were used in these studies.

Gene Symbol	Forward	Reverse
36B4	GAAACTGCTGCCTCACATCCG	GCTGGCACAGTGACCTCACACG
UCP1	ACTGCCACACCTCCAGTCATT	CTTTGCCTCACTCAGGATTGG
PGC1α	AGCCGTGACCACTGACAACGAG	GCTGCATGGTTCTGAGTGCTAAG
Dio2	CAGTGTGGTGCACGTCTCCAATC	TGAACCAAAGTTGACCACCAG
ELovl3	TCCGCGTTCTCATGTAGGTCT	GGACCTGATGCAACCCTATGA

## Supplementary Materials

Supplementary materials can be found at https://www.mdpi.com/1422-0067/20/10/2456/s1.

## Abbreviations

MOTS-c	Mitochondrial ORF of the twelve S c
UCP1	Uncoupled protein 1
PGC1α	PPARG coactivator 1 alpha
Dio2	Iodothyronine deiodinase 2
Elovl3	ELOVL fatty acid elongase 3
TGs	Triglycerides
