# Supplementary material for: Mitochondrial-Derived Peptide MOTS-c Increases Adipose Thermogenic Activation to Promote Cold Adaptation
Source: Int J Mol Sci. 2019 May 17;20(10):2456. doi: 10.3390/ijms20102456 (PMC6567243; doi:10.3390/ijms20102456)
Supplement: Supplementary file 1 [file ijms-20-02456-s001.pdf]

International Journal of Molecular Sciences

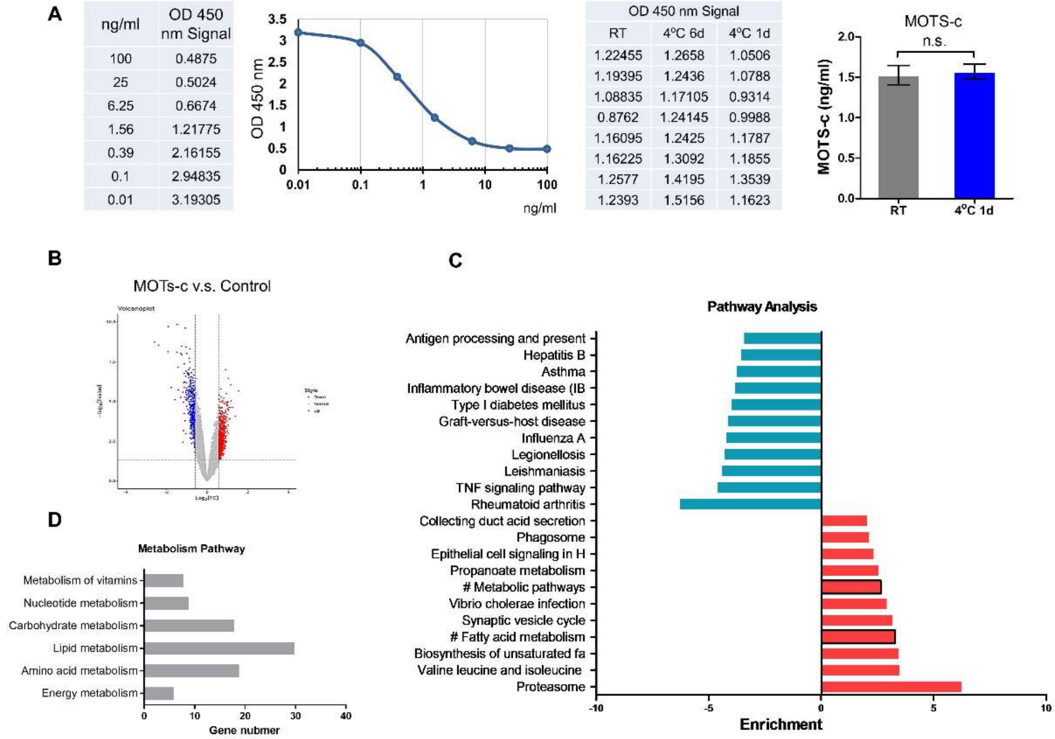

Figure S1. MOTS-c involves in regulating metabolic pathway. (**A**) The plasma level of MOTS-c following 4 °C exposure and the standard curve and 450 nm absorption signaling are shown (n = 8 per group). (**B**) Significant changed genes shown in volcano plot (MOTS-c v.s. Control, fold change > 1.5, p value < 0.05, n = 3); (**C**) KEGG Pathway remarkably enriched in MOTS-c treated group, and Metabolic pathways and Fatty acid metabolism were highlighted by "#". (**D**) Gene numbers in different metabolic signaling pathway.

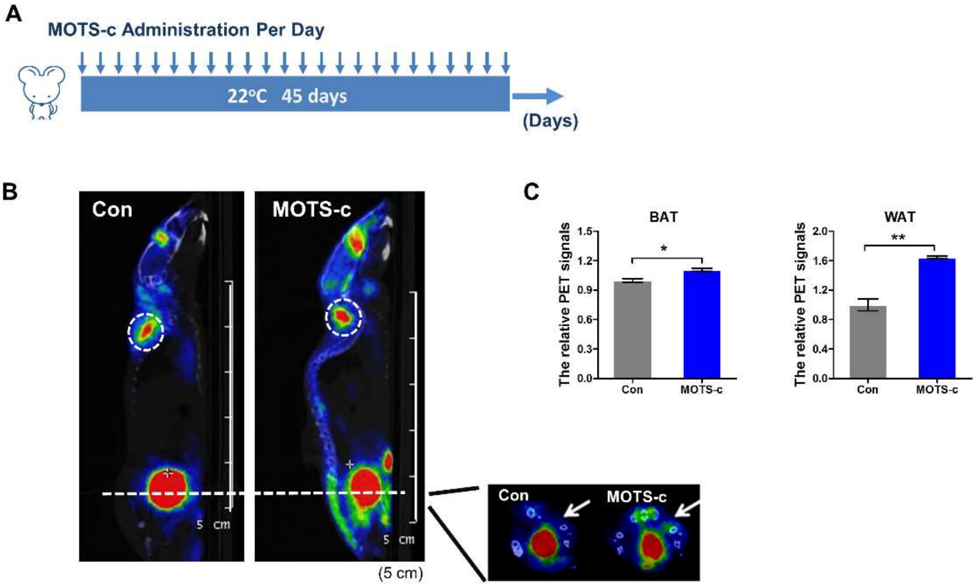

Figure 2. MOTS-c increases the activation of brown and white adipose under room temperature. (**A**) Experimental design for the study in MOTS-c administration under room temperature. (**B**) PET/ CT image after injection of 18F-FDG into mice (15 weeks) treated with normal saline (Con) and MOTS-c (5 mg/kg, per day) for 45 days in sagittal view. (scale bar = 5 cm). Anatomical site within white circle represent the interscapular BAT. Anatomical site marked by white dotted line represent the

subcutaneous WAT, shown in coronal view. (**C**) The relative PET signals showed the activity of BAT and WAT. All data are represented as mean ± SEM. Differences between the two groups were determined by a two-tailed Student's *t*-test. * $p < 0.05$，** $p < 0.01$.
